# A Pilot Study of Host Genetic Variants Associated with Influenza-associated Deaths among Children and Young Adults^1^

**DOI:** 10.3201/eid1712.111002

**Published:** 2011-12

**Authors:** Jill M. Ferdinands, Amy M. Denison, Nicole F. Dowling, Heather A. Jost, Marta L. Gwinn, Lindy Liu, Sherif R. Zaki, David K. Shay

**Affiliations:** Centers for Disease Control and Prevention, Atlanta, Georgia, USA

**Keywords:** viruses, influenza, children, genetics, polymorphism, death, Staphylococcus aureus

## Abstract

We compared the prevalence of 8 polymorphisms in the tumor necrosis factor and mannose-binding lectin genes among 105 children and young adults with fatal influenza with US population estimates and determined in subanalyses whether these polymorphisms were associated with sudden death and bacterial co-infection among persons with fatal influenza. No differences were observed in genotype prevalence or minor allele frequencies between persons with fatal influenza and the reference sample. Fatal cases with low-producing *MBL2* genotypes had a 7-fold increased risk for invasive methicillin-resistant *Staphylococcus aureus* (MRSA) co-infection compared with fatal cases with high- and intermediate-producing *MBL2* genotypes (odds ratio 7.1, 95% confidence interval 1.6–32.1). Limited analysis of 2 genes important to the innate immune response found no association between genetic variants and fatal influenza infection. Among children and young adults who died of influenza, low-producing *MBL2* genotypes may have increased risk for MRSA co-infection.

## Abstract 2

Low-producing *MBL2* genotypes may have increased risk for MRSA co-infection.

It is unknown why some apparently healthy persons become severely ill after influenza infection while others infected by the same strain remain asymptomatic or become only mildly ill. The presence of neutralizing antibody to a specific influenza strain is protective, and certain chronic medical conditions increase the risk for severe outcomes of influenza infections, but the risk factors for influenza-associated deaths among previously healthy persons remain largely unknown (*1*).

Infectious disease mortality risk has a heritable component; children of parents who died of an infectious disease are ≈6× more likely to die of an infectious cause compared with the general population (*2*). A recent large family study that used genealogic databases found an elevated risk for influenza death among relatives of persons who died of influenza (*3*). By comparing the influenza mortality rate for relatives of persons who died of influenza with the influenza mortality rate for relatives of spouses of persons who died, the authors showed that the increased risk was not explained by shared exposure to influenza virus and thus may have a genetic component. However, to our knowledge, no published studies have examined the association between specific host genetic variants and severe influenza disease outcomes.

To address the paucity of research on host genomics and influenza, the Centers for Disease Control (CDC) convened a meeting of experts in 2007 to solicit opinions on how to explore the role of host genomics in public health activities for influenza conducted by the agency. A study of host genomic factors related to severe influenza outcomes in children was recommended as an activity that CDC was well positioned to pursue. This article reports the findings of the study implemented in response to that recommendation.

We conducted a hypothesis-generating pilot study to examine if host genetic variants were associated with fatal influenza virus infection by comparing prevalence of selected host genetic variants among children and young adults who died of influenza with population-based prevalence estimates. We focused on 8 single-nucleotide polymorphisms (SNPs) in 2 candidate genes important in the innate immune response to influenza infection and for which national prevalence estimates were available: the gene for tumor necrosis factor superfamily, member 2 (official symbol *TNF*) and the mannose-binding lectin gene (official symbol *MBL2*).

## Methods

### Study Population

Because influenza-associated deaths in children, but not adults, are nationally reportable in the United States, most cases in this study were pediatric cases reported to CDC through the Influenza-associated Pediatric Mortality Surveillance system. This system requires state public health authorities to report to CDC any influenza-associated death among persons <18 years old that occurred within their jurisdiction. Information collected by this surveillance system constitutes the primary phenotypic information used in this study and includes underlying health status and chronic medical conditions, influenza vaccination status, clinical course and features, and results of microbiologic and virologic testing. Reporting to this surveillance system does not require submission of tissue samples; however, CDC routinely receives tissue samples for a subset of fatal pediatric influenza cases for diagnostic confirmation. For some cases, medical records and autopsy reports provided additional information.

A total of 442 influenza-associated deaths among children (<18 years old) and young adults (18–40 years old) residing in the United States were reported to CDC for the 1998–99 through 2007–08 influenza seasons; of these, 105 cases with laboratory-confirmed influenza infection had sufficient tissue specimens available for DNA extraction and constitute the analytic dataset for this study. Fatal influenza cases were considered laboratory confirmed if a positive test result for influenza by viral culture, immunohistochemical analysis, or reverse transcription PCR (RT-PCR) had been documented. These represented 1) fatal pediatric cases reported to CDC during the 2003–04 influenza season when CDC conducted surveillance for influenza-associated pediatric deaths as part of an emergency response effort; 2) fatal pediatric cases identified through national surveillance since 2004 when pediatric influenza–associated death was made nationally notifiable in the United States; or 3) fatal cases of influenza among young adults at any point in time or among children before 2003 whose case reports and specimens were received by the CDC Infectious Diseases Pathology Branch on a case-by-case basis.

### Genotyping

To obtain DNA for genotyping, a 10-μm section from blocks containing formalin-fixed, paraffin-embedded tissues was deparaffinized with xylene and washed twice with absolute ethanol. After residual ethanol evaporated, tissues were digested overnight at 56°C in 200 µL Buffer PKD with 20 µL proteinase K (QIAGEN, Valencia, CA, USA). Extraction of the supernatant was completed with an EZ1 DNA Tissue Kit or a MagAttract DNA Mini M48 Kit (QIAGEN), with DNA eluted into a final 100-µL volume. DNA quality was assessed with a human RNase P real-time PCR in 25-μL volumes by using Agilent Brilliant II QPCR Master Mix as described (*4*). Validated TaqMan assays were used to genotype each SNP (protocols, primers, and probes available at http://snp500cancer.nci.nih.gov). Each 25-μL real-time PCR consisted of 12.5 μL of TaqMan Universal PCR Master Mix (Applied Biosystems, Foster City, CA, USA), 900 nmol of assay-specific primer, 200 nmol of assay-specific probe, and 5 μL of DNA. All controls (extraction blanks, no template controls, and positive controls for each genotype used at 5 ng per PCR; Coriell Institute for Medical Research, Camden, NJ, USA) and unknown samples were assayed in duplicate. Thermal cycling conditions consisted of 1 cycle at 50°C for 2 min, 1 cycle at 95°C for 10 min, and 50 cycles of 92°C for 30 s and 60°C for 1 min. Data were collected during the annealing plateau.

### Genotype Definitions

For *TNF*, we examined 3 promoter SNPs: −308G>A (rs1800629), −238G>A (rs361525), and −555G>A (rs1800750) (*5**,**6*); we were unable to infer *TNF* haplotypes. For *MBL2*, we examined 5 SNPS, 3 in the coding region of exon 1 and 2 in the promoter region. The 3 structural SNPs in *MBL2* that we examined encode variant alleles known as *D* (codon 52, rs5030737), *B* (codon 54, rs1800450), and *C* (codon 57, rs1800451); the wild-type is *A* (*7**,**8*). These variants are typically pooled and designated as the *O* allele. The *MBL2* genotype *A/A* refers to wild-type homozygotes, *A/O* refers to heterozygotes, and *O/O* refers to homozygotes or compound heterozygotes. Promoter polymorphisms at positions −550 (*H/L* variant, rs11003125) and −221 (*X/Y* variant, rs7096206) encode variants that mediate *MBL2* expression. Case-patients were classified as low, intermediate, or high producers of MBL on the basis of their structural and promoter variants (referred to as a “truncated haplotype”) (*7*). Case-patients homozygous or compound heterozygous for any of the 3 variant structural alleles and case-patients with a variant structural allele on 1 chromosome and the *X* variant on the other were categorized as low MBL producers. Case-patients homozygous for the wild-type structural allele were categorized as high MBL producers except for those also homozygous for the *X* variant, who were classified as intermediate MBL producers on the basis of evidence that possession of the *X/X* promoter genotype significantly down-regulates MBL production (*9*). Case-patients with the *YA/O* genotype were classified as intermediate MBL producers on the basis of analyses indicating that this genotype confers intermediate levels of functional MBL (*9*). For some analyses, the intermediate and high producers were combined into 1 group and compared with MBL low-producers.

### Reference Sample

The prevalence of genetic variants among cases was compared with population-based prevalence estimates for the same genetic variants for the 12–19-year age group available from the National Health and Nutrition Examination Survey (NHANES) III CDC–National Cancer Institute Collaborative Genomics Project databank (*10*). NHANES is a nationally representative survey of the US population conducted by CDC’s National Center for Health Statistics. During the second phase of NHANES III (1991–1994), leukocytes from participants were used to create a DNA bank maintained by the CDC National Center for Environmental Health that contains specimens from >7,000 participants, including ≈1,200 children. To our knowledge, the NHANES DNA bank is the only currently available source of nationally representative prevalence estimates for genetic variants among US residents. The 12–19-year age group is the youngest age group available in the NHANES DNA bank.

### Variable Definitions

Cases were stratified by presence or absence of any chronic medical conditions in the patients known to increase the risk for influenza-associated complications (including moderate to severe developmental delay; hemoglobinopathy, immunosuppressive disorders, asthma or reactive airway disease, diabetes mellitus, history of febrile seizures, seizure disorder, cystic fibrosis, or cardiac, renal, chronic pulmonary, metabolic, or neuromuscular disorders) (*11*). Case-patients without chronic medical conditions were classified as “previously healthy.” Case-patients who were admitted to an inpatient ward or intensive care unit were classified as “hospitalized.” Length of illness was defined as the duration of time between the reported date of illness onset and death. Case-patients with length of illness <3 days were classified as having “sudden death.” Bacterial co-infection was defined as at least 1 positive culture for a bacterial pathogen from a normally sterile site (e.g., blood, cerebrospinal fluid).

### Statistical Analyses

Minor allele frequencies between groups were compared with a test of binomial proportions. The null hypothesis was that there was no difference in minor allele frequency between the cases and the reference sample. A priori groups examined in subgroup analyses included previously healthy case-patients, case-patients <5 years old, case-patients with invasive bacterial co-infection, and case-patients with sudden death. Differences in length of illness were evaluated with the Kaplan-Meier estimator with differences tested with the log-rank statistic. Tests of significance were based on a 2-sided test with α = 0.05. Tests of departure from Hardy-Weinberg equilibrium for the reference sample have been published (*10*). Analyses were conducted in SAS version 9.2 (SAS Institute, Cary, NC, USA).

### Human Subjects

This study was exempted from institutional review board review for approval of human subjects research. Data were obtained only from deceased case-patients, and reference sample data were used only in a de-identified and aggregate manner.

## Results

### Participant Characteristics

Of 442 cases of fatal influenza in children and young adults reported to CDC during the 1998–99 through 2007–08 influenza seasons, 105 (24%) cases had available autopsy specimens with sufficient DNA for genotyping. Case-patient characteristics are summarized in Table 1. Genotyped case-patients had a median age of 6.0 years (range 1 month–40 years) and 52% were female. Sixty-one percent of case-patients were white, and 17% were black. Seventy-four percent of cases occurred during 3 influenza seasons: 2003–04 (31%), 2006–07 (21%), and 2007–08 (22%). Eighty-one (77%) of 105 case-patients were infected with influenza A and 24 (23%) with influenza B. There were no significant differences in the distribution of influenza types by season between cases and the national pattern of types found in the US viral surveillance system (data not shown).

**Table 1:** Characteristics of and course of illness for in children and young adults who died of influenza, reported to the Centers for Disease Control and Prevention during influenza seasons 1998–99 through 2007–08, United States*

Characteristic	Genotyping status		All, n = 442
	Genotyped, n = 105	Not genotyped, n = 337	
Patient demographics			
Median age, y (range)†	6.0 (0.1–40.0)	4.0 (0.0–17.3)	4.3 (0.0–40.0)
Female sex	55 (52)	158 (47)	213 (48)
Race			
White	64 (61)	208 (62)	272 (62)
Black	18 (17)	62 (18)	80 (18)
Asian	7 (7)	17 (5)	24 (5)
Other	3 (3)	9 (3)	12 (3)
Unknown/missing	13 (12)	41 (12)	54 (12)
Influenza season			
1998–99	1 (1)	0	1 (<1)
1999–00	0	0	0
2000–01	1 (1)	0	1 (<1)
2001–02	2 (2)	0	2 (<1)
2002–03	10 (10)	0	10 (2)
2003–04	33 (31)	121 (36)	154 (35)
2004–05	7 (7)	44 (13)	51 (12)
2005–06	6 (6)	42 (12)	48 (11)
2006–07	22 (21)	60 (18)	82 (19)
2007–08	23 (22)	70 (21)	93 (21)
Influenza type			
A	81 (77)	233 (69)	314 (71)
B	24 (23)	65 (19)	89 (20)
A/B not distinguished	0	36 (11)	36 (8)
Unknown/missing	0	3 (<1)	3 (<1)
Preexisting medical condition‡	29 (28)	205 (61)	234 (53)
Vaccinated season of death§	7 (7)	54 (16)	61 (14)
Sudden death†	33 (31)	74 (22)	107 (24)
Length of illness, d, median (range)‡	3.0 (0–15)	5.0 (0–194)	5.0 (0–194)
Location of death‡			
ICU/inpatient	28 (27)	216 (64)	244 (55)
Emergency department	25 (24)	48 (14)	73 (17)
Outside hospital	36 (34)	73 (22)	109 (25)
Unknown/missing	16 (15)	0	16 (4)
Mechanical ventilation†	41 (39)	216 (64)	257 (58)
ICU admission‡	28 (27)	165 (49)	193 (44)
Invasive bacterial co-infection	22 (21)	79 (23)	101 (23)
Invasive MRSA co-infection	8 (8)	24 (7)	32 (7)
Pneumonia evident on chest radiograph†	23 (22)	154 (46)	177 (40)

*Values are no. (%) patients except as indicated. A χ^2^ test of difference in proportion was used to test differences in proportion between genotyped and ungenotyped cases except for patient age, season, and length of illness, for which the Kruskal-Wallis nonparametric test of difference was used. A total of 442 possible or confirmed influenza-related deaths among children (<18 years old) and young adults (18–40 years old) were reported to the Centers for Disease Control and Prevention (CDC) from 1998–99 through 2007–08. Of these, 105 case-patients had laboratory-confirmed influenza infection and sufficient tissue specimen available at CDC for genotyping. ICU, intensive care unit; MRSA, methicillin-resistant *Staphylococcus aureus*. †p<0.05. ‡p<0.001. §p<0.01.

Compared with case-patients who were not genotyped, the 105 case-patients with DNA available for genotyping were slightly older (median age 6 years vs. 4 years; p<0.05), less likely to have had a preexisting medical condition (28% vs. 61%; p<0.001), and less likely to have been vaccinated for influenza during the season of death (7% vs. 16%; p<0.01). Case-patients genotyped were more likely to have experienced sudden death (31% vs. 22%; p<0.05) and to have died before reaching medical care (34% vs. 22%; p<0.001). It is not surprising that case-patients with sudden death were more likely to have undergone autopsy and, hence, to have had tissues available for DNA extraction. Genotyped case-patients were less likely to have had pneumonia evident on chest radiograph (22% vs. 46%; p<0.05) and about equally likely to have had invasive bacterial co-infection (21% vs. 23%; not significant), but differences in these characteristics are difficult to interpret because genotyped case-patients were less likely to have received medical care for their illnesses (presumably because of a greater frequency of sudden death).

### Genotyping Results

**Figure 1:**
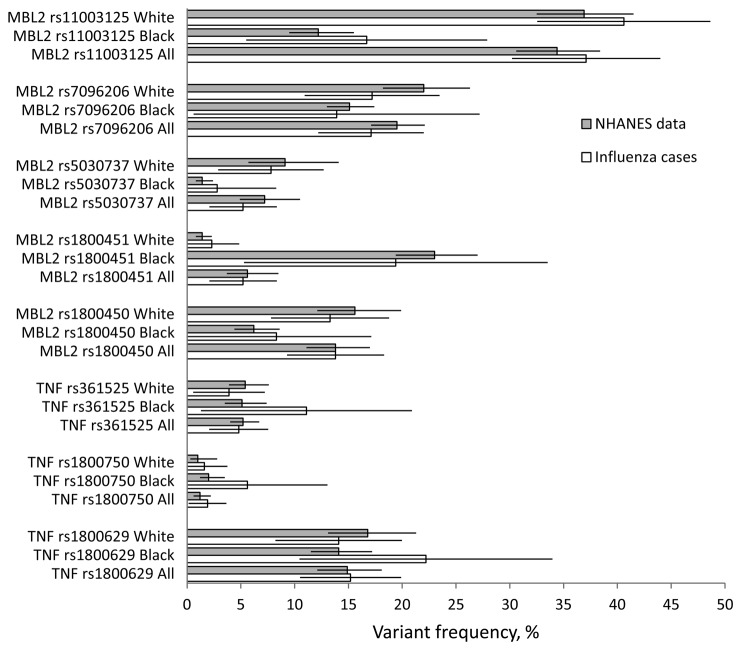
Variant frequency and 95% confidence intervals for fatal influenza cases compared with the NHANES reference group for 8 single-nucleotide polymorphisms. Allele frequency did not differ significantly between cases and the reference group for any single-nucleotide polymorphism. NHANES, National Health and Nutrition Examination Survey. Error bars represent confidence intervals.

Genotype and minor allele frequencies among case-patients are summarized in Table 2. Minor allele frequencies comparing case-patients to the NHANES reference sample are shown in Figure 1.

**Table 2:** Genotype and minor allele frequencies among 105 children and young adults who died of influenza, United States, 1998–99 through 2007–08 influenza seasons*

Gene	Variant	Genotype	Genotype frequency, no. (%) case-patients			
			All, n = 105	White, n = 64	Black, n = 18	Asian, n = 7
*TNF*	rs1800629	−308GG	74 (70)	47 (73)	10 (56)	6 (86)
		−308GA	30 (29)	16 (25)	8 (44)	1 (14)
		−308AA	1 (1)	1 (2)	0	0
		A allele	0.152	0.141	0.222	0.071
	rs1800750	−555GG	101 (96)	62 (97)	16 (89)	7 (100)
		−555GA	4 (4)	2 (3)	2 (11)	0
		−555AA	0	0	0	0
		A allele	0.019	0.016	0.056	0
	rs361525	−238GG	95 (90)	59 (92)	14 (78)	6 (86)
		−238GA	10 (10)	5 (8)	4 (22)	1 (14)
		−238AA	0	0	0	0
		A allele	0.048	0.039	0.111	0.071
*MBL2*	rs1800450 (*B* variant)	27GG	77 (73)	47 (73)	15 (83)	5 (71)
		27GA	27 (26)	17 (27)	3 (17)	2 (29)
		27AA	1 (1)	0	0	0
		A allele	0.138	0.133	0.083	0.143
	rs1800451 (*C* variant)†	18GG	95 (90)	61 (95)	12 (67)	7 (100)
		18GA	9 (9)	3 (5)	5 (28)	0
		18AA	1 (1)	0	1 (6)	0
		A allele	0.052	0.023	0.194	0
	rs5030737 (*D* variant)	34CC	95 (90)	55 (86)	17 (94)	7 (100)
		34CT	9 (9)	8 (13)	1 (6)	0
		34TT	1 (1)	1 (2)	0	0
		T allele	0.052	0.078	0.028	0
	rs7096206 (X/Y variant)	−221CC	71 (68)	43 (67)	14 (78)	4 (57)
		−221CG	32 (30)	20 (31)	3 (17)	3 (43)
		−221GG	2 (2)	1 (2)	1 (6)	0
		G allele	0.171	0.172	0.139	0.214
	rs11003125 (H/L variant)	−550CC	43 (41)	21 (33)	12 (67)	2 (29)
		−550CG	46 (44)	34 (53)	6 (33)	4 (57)
		−550GG	16 (15)	9 (14)	0	1 (14)
		G allele	0.371	0.406	0.167	0.429
	Pooled structural variants	AA	59 (56)	37 (58)	8 (44)	5 (71)
		AO	41 (39)	24 (38)	9 (50)	2 (29)
		OO	5 (5)	1 (2)	1 (6)	0
	Truncated haplotypes	YA/YA	36 (34)	21 (33)	5 (28)	3 (43)
		XA/YA	21 (20)	15 (23)	2 (11)	2 (29)
		XA/XA	2 (2)	1 (2)	1 (6)	0
		YA/O	30 (29)	19 (30)	8 (44)	1 (14)
		XA/O	11 (10)	5 (8)	1 (6)	1 (14)
		O/O	5 (5)	3 (5)	1 (6)	0
	MBL production	Low	16 (15)	8 (13)	2 (11)	1 (14)
		Intermediate	32 (31)	20 (31)	9 (50)	1 (14)
		High	57 (54)	36 (56)	7 (39)	5 (71)

*Cases include 16 patients with missing/unknown or other race. Per common designation, for *MBL2*, A refers to the wild-type structural allele, O refers to any of the 3 variant structural alleles, and O/O refers to any combination of structural variant alleles. Low producers included truncated haplotypes O/O and XA/O. *MBL2*, mannose-binding lectin gene. †p<0.01 by Fisher exact test of difference in proportion across racial group.

### TNF

No statistically significant differences were observed in minor allele frequencies or genotype prevalence between the case-patients and the NHANES reference sample for the 3 *TNF* variants with all case-patients examined together or with black and white racial groups examined separately. Length of illness was shorter among case-patients with the *TNF* rs1800750 AG genotype than among those with the GG genotype (median length of illness, 1 day vs. 3 days, p = 0.001); no case-patients had the AA genotype. The estimated odds ratio for sudden death was 15.0 for case-patients with the AG genotype compared with case-patients with the GG genotype (p = 0.04 by Fisher exact test). No significant associations were found between any *TNF* variant examined and bacterial co-infection.

### *MBL*2

**Figure 2:**
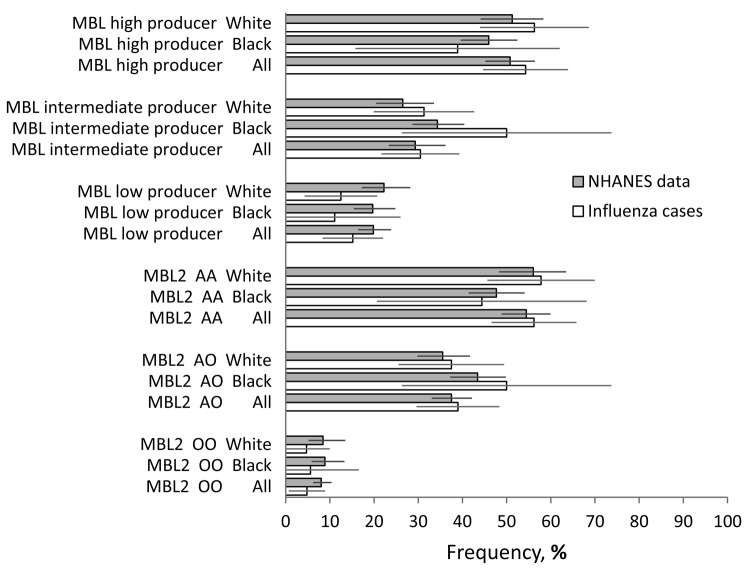
Frequency and 95% confidence intervals for fatal influenza cases (n = 105) compared with the NHANES reference group for pooled structural genotypes (AA/AO/OO) of *MBL*2 and categorization of MBL production on the basis of genotype (low/intermediate/high). Frequencies did not differ significantly between cases and the reference group. MBL, mannose-binding lectin; NHANES, National Health and Nutrition Examination Survey; *MBL*2, mannose-binding lectin gene. Error bars represent confidence intervals.

No statistically significant differences were observed in minor allele frequencies for the 5 *MBL2* SNPs examined (Figure 1) or the prevalence of pooled *MBL2* genotypes (Figure 2) between the case-patients and the NHANES reference sample with all case-patients examined together or with black and white racial groups examined separately. In a subgroup analysis, the minor allele frequency of rs5030737 was significantly less common among case-patients <5 years old than in the reference sample (2% vs. 7.2%; p = 0.02). Among low producers of MBL, we observed an estimated odds ratio of 7.1 (95% confidence interval [CI] 1.6–32.1) for invasive methicillin-resistant *Staphylococcus aureus* (MRSA) co-infection compared with case-patients with high or intermediate MBL production, according to *MBL2* genotype (p = 0.02 by Fisher exact test; Table 3). Low-producing *MBL2* genotypes were also associated with an approximate 3-fold increased risk for bacterial co-infection in general and with *S. aureus* infection overall, but these associations did not reach statistical significance. Characteristics of case-patients with invasive MRSA co-infection are shown in Table 4.

**Table 3:** Associations between *MBL2* haplotypes and invasive bacterial co-infection in fatal influenza in children and young adults, United States, 1998–99 through 2007–08 influenza seasons*

Outcome	Estimated odds ratio (95% CI)		
	Low producing *MBL2* haplotype†	Intermediate or high producing *MBL2* haplotype‡	p value
MRSA, n = 8	7.1 (1.6–32.1)	Referent	0.02
*Staphylococcus aureus*, n = 13	3.0 (0.8–11.2)	Referent	0.11
Bacterial co-infection, n = 22	2.7 (0.9–8.6)	Referent	0.10

*MBL2, mannose-binding lectin gene; MRSA, methicillin-resistant *Staphylococcus aureus*. †includes *MBL2* haplotypes O/O and XA/O. ‡includes *MBL2* intermediate producer haplotypes XA/XA and YA/O and high producer haplotypes XA/YA and YA/YA.

**Table 4:** Characteristics of 8 children and young adults who died of influenza and had invasive methicillin-resistant *Staphylococcus aureus* co-infection, United States, 1998–99 through 2007–08 influenza seasons

Patient age, y/sex	Season	Influenza type	Previous conditions	Sudden death*	*MBL2* haplotype†	MBL production haplotype‡	*MBL2* structural variant
29/M	2004–05	A	None	Yes	YA/O	Intermediate	rs1800450 A/G
14/M	2006–07	A	None	No	XA/YA	High	None
11/F	2006–07	B	None	Yes	YA/YA	High	None
13/M	2006–07	B	None	Yes	XA/O	Low	rs1800450 A/G
12/F	2006–07	B	None	No	O/O	Low	rs1800450 A/A
8/F	2007–08	A	Asthma	Yes	XA/O	Low	rs1800450 A/G
18/M	2007–08	A	Asthma	Yes	XA/O	Low	rs1800450 A/G
32/M	2007–08	B	Asthma	No	XA/YA	High	None

*Sudden death is defined as death occurring less than 3 days after symptom onset. *MBL2*, mannose-binding lectin gene. †Per common designation for *MBL2*, A refers to the wild-type structural allele, O refers to any of the 3 variant structural alleles, and O/O refers to any combination of structural variant alleles. Y refers to the wild-type allele and X to the variant allele at promoter locus rs7096206 (the X/Y variant). ‡Low mannose-binding lectin (MBL) producers included *MBL2* haplotypes O/O and XA/O. Intermediate producers included *MBL2* haplotypes XA/XA and YA/O. High producers included *MBL2* haplotypes XA/YA and YA/YA.

## Discussion

We found no significant differences in allele frequencies or genotype prevalence for variants in the *TNF* and *MBL2* genes between fatal influenza cases in patients <40 years old and a nationally representative reference sample. However, among the case-patients who died, most of whom died in childhood, variants of *MBL2* responsible for low production of MBL were associated with MRSA co-infection. This observation should be viewed cautiously as a hypothesis for further exploration, given the small number of case-patients with MRSA in our study (n = 8). This finding is consistent with results from previous studies that found associations between MBL insufficiency (defined by genotype) and respiratory infection in children (*12**–**14*), severe and fatal sepsis (*9**,**15**–**17*), and systemic inflammatory response syndrome in children (*18*).

TNF is a potent proinflammatory cytokine produced early in the innate immune response to infection that promotes a wide range of immunologic responses. Excessive systemic TNF is responsible for many symptoms of clinical infection and may lead to fatal complications. Studies have demonstrated a significant genetic contribution to circulating TNF levels, with 50%–60% of variance in TNF levels genetically determined (*19**–**21*). The most studied SNP is at position −308 (rs1800629), with the A allele associated with 20%–40% greater TNF production (*22**–**24*) and with susceptibility to and severity of numerous infectious diseases (*20**,**22**,**25**,**26*). Carriage of the A allele at the −238 position (rs361525) also has been associated with a variety of diseases (*20**,**22*).

MBL, another key component of the innate immune system, is a soluble protein of the collectin family that binds to microbial surfaces and promotes phago-opsonization directly and indirectly by activating the lectin complement pathway. Low serum MBL levels are common and associated with an increased risk for a variety of infections and autoimmune diseases (*15**,**27**–**29*), including acute respiratory infection in young children (*12*). MBL levels are strongly influenced by genetic factors, with >75% of variation in MBL levels explained by a small number of polymorphisms in the *MBL2* gene (*30*). Variant proteins are unstable and of lower oligomeric form, which decreases affinity for microbial ligands and complement-activating ability. Each variant produces significantly reduced serum MBL levels.

MBL has been shown to strongly bind *S. aureus* (*31*) and susceptibility to fatal *S. aureus* infection due to MBL deficiency has been convincingly demonstrated in murine models (*32*). Phase I clinical trials of MBL replacement therapy indicate that this therapy is well tolerated and effective at improving MBL deficiency in healthy persons (*33*). Reports of MBL replacement therapy administered to severely ill persons (*34**–**36*) or to patients with *S. aureus* sepsis (*37*) suggest that therapy can improve clinical conditions, although results of these studies were mixed, and in some cases, clinical improvements were temporary. The clinical implications of MBL replacement therapy for influenza treatment or prevention are unknown.

Among persons with fatal cases, we observed an increased risk for sudden death in carriers of the variant allele of *TNF* rs1800750. We are unaware of previous literature reporting a similar association; there is no obvious biologic mechanism to explain the finding. The *TNF* rs1800750 variant is in linkage disequilibrium with other *TNF* variants (http://pga.gs.washington.edu), some of which (including *TNF* rs361525) have been associated with increased TNF serum levels. Therefore, it is possible that the observed association may be due to linkage disequilibrium with unmeasured polymorphisms that are the causal variants, and more exhaustive analysis of *TNF* variants is worthy of future study.

A strength of this study is its use of a cohort of case-patients particularly well-suited for investigation of potential host genetic risk factors—these case-patients died with active influenza infections, yet were predominantly children and young adults without severe preexisting medical conditions. In such a group, other factors associated with severe influenza are less likely to obscure possible genetic associations. An additional strength was access to postmortem lung tissue for immunohistochemistry and/or RT-PCR confirmation of influenza infection.

We recognize that this study has several limitations. Although the study cohort is, to our knowledge, the largest sample of fatal influenza cases in children and young adults, the analysis has limited statistical power to detect associations because of small sample sizes, especially when examining subsamples. We had access to limited information about racial and ethnic background of case-patients. Clinical data were obtained primarily from a US surveillance system and were not validated with medical chart review. Although we were able to infer truncated haplotypes for *MBL2*, haplotype information for *TNF* was unavailable. Despite these shortcomings, the possibility that specific variants of the *MBL2* gene known to influence serum MBL levels appear to be associated with severe bacterial co-infection is an intriguing finding deserving of additional study, especially given the prevalence of co-infection among case-patients who died of pandemic (H1N1) 2009 virus infection (*38*) and observations that children co-infected with influenza and *S. aureus* may have higher case-fatality rates (*39*).

That we observed a stronger relationship between low-producing MBL genotypes and MRSA infection than between those genotypes and *S. aureus* infection in general is puzzling. We are unaware of an obvious physiologic explanation for why low MBL would predispose more strongly to infection with methicillin-resistant versus methicillin-sensitive *S. aureus*. One possibility is that MRSA is a marker for other strain characteristics. For example, such an association could arise if MRSA infections were predominantly the USA300 strain while other *S. aureus* infections were predominantly the USA100 strain. Unfortunately, we do not have data on *S. aureus* genetic strain types. We also found that of the 4 fatal influenza cases in which patients had both MRSA co-infection and low-producing MBL genotypes, 2 patients reportedly also had asthma. It is well-established that asthma increases the risk for serious complications of influenza, and although we know of no evidence suggesting that low-producing MBL genotypes are associated with increased risk for asthma (*40*), this finding may be worth further exploration in future studies.

Our findings suggest several opportunities for additional influenza-related research. An obvious next step is examination of all functional variants of the *MBL2* gene in conjunction with gene expression and functional assays in a larger group of severely ill influenza case-patients with sufficiently detailed clinical data to define important phenotypes (e.g., MRSA co-infection). Interest in association studies of rare variants, the availability of new sequencing technologies that dramatically decrease the cost of sequencing, and access to reference human sequence data suggest that investigating rare variants in candidate genes (including *MBL2* and *TNF*) and their functional effects may be a promising avenue of research. Large-scale genotyping of a sample of case-patients to look for common variants by using methods such as genomewide association studies may be possible if a network of collaborators capable of pooling a sufficient number of case-patients is developed. Recent initiatives such as the Genome-based Research and Population Health International Network (www.graphint.org/ver2) are aimed at encouraging such networks. Given the rapid acceleration in laboratory technologies, enhancement in bioinformatics methods and capacity, and trends toward collaborative research within large consortia, exploration of the role of host genomic factors in serious illness associated with influenza and other viral pathogens is increasingly feasible. We believe that host genomics is a promising area for future research regarding who is at risk for severe complications of acute infectious diseases, including influenza.
